# Regional differences in prevalence, awareness, treatment and control of hypertension in Poland - comparison of two national multi-center health surveys: WOBASZ and WOBASZ II

**DOI:** 10.1371/journal.pone.0331677

**Published:** 2025-10-27

**Authors:** Małgorzata Wierzowiecka, Justyna Marcinkowska, Andrzej Pająk, Magdalena Kozela, Jerzy Piwoński, Wojciech Drygas, Magdalena Kwaśniewska, Tomasz Zdrojewski, Krystyna Kozakiewicz, Andrzej Tykarski, Arkadiusz Niklas, on behalf of WOBASZ II investigators

**Affiliations:** 1 Department of Hypertension, Angiology and Internal disease, University of Medical Science, Poznan, Poland; 2 Department of Computer Science and Statistics, Poznan University of Medical Science, Poznan, Poland; 3 Chair of Epidemiology and Population Studies, Institute of Public Health, Faculty of Health Sciences, Jagiellonian University Medical College, Krakow, Poland; 4 Department of Epidemiology, Cardiovascular Disease Prevention and Health Promotion, Institute of Cardiology, Warsaw, Poland; 5 Calisia University, Kalisz, Poland; 6 Department of Social and Preventive Medicine, Medical University of Lodz, Poland; 7 Department of Arterial Hypertension and Diabetology, Medical University of Gdansk, Poland; 8 Department of Cardiology, Medical University of Silesia, Katowice, Poland; University of Oxford, UNITED KINGDOM OF GREAT BRITAIN AND NORTHERN IRELAND

## Abstract

**Introduction:**

Data on regional differences in the current prevalence of hypertension in Poland remain limited.

**Objectives:**

This study aimed to assess the prevalence, awareness, treatment, and control of hypertension among Polish adults in 2013–2014 (WOBASZ II study) and to compare these parameters with data from 2003–2005 (WOBASZ study).

**Patients and Methods:**

The study was conducted in two independent, representative samples. The WOBASZ II study included 3,406 women and 2,757 men, while the WOBASZ study examined 7,783 women and 6,972 men.

**Results:**

The greatest increase in hypertension prevalence over 10 years was observed among men from Świętokrzyskie Voivodeship (39.7% vs. 51.8%) and women from Łódzkie Voivodeship (32.7% vs. 44%). Increases in hypertension awareness were noted in men from Wielkopolskie Voivodeship (50.8% vs. 59.5%) and women from Świętokrzyskie Voivodeship (65.7% vs. 77%). Treatment rates rose in men from Warmińsko-Mazurskie Voivodeship (16.8% vs. 40.3%) and women from Opolskie Voivodeship (24.7% vs. 44.4%). Improvements in hypertension control were seen in men from Wielkopolskie Voivodeship (3.5% vs. 13.3%) and women from Lubuskie Voivodeship (5.6% vs. 27.4%).

**Conclusions:**

Pronounced differences were observed in the prevalence, awareness, treatment, and control of arterial hypertension among voivodeships, highlighting the necessity of region-specific public health interventions and resource allocation to effectively manage hypertension. Additionally, changes observed over the decade from 2003/04–2013/14 highlight evolving epidemiological trends and demonstrate the impact of such interventions on hypertension management.

## Introduction

Hypertension is one of the most important modifiable risk factors for cardiovascular disease. Over the past decade, the prevalence of hypertension has decreased by 2.6% in high-income countries, while it has increased by 7.7% in low- and middle-income countries [1]. In 2000, 26.4% of adults were diagnosed with hypertension. From 2000 to 2025, the estimated number of people with hypertension will increase from about 972 million to 1.56 billion [2]. Between 2000 and 2010, awareness of hypertension standardized for age (58.2% vs. 67.0%), treatment (44.5% vs. 55.6%) and control (17.9% vs. 28.4%) increased in high-income countries, whereas awareness (32.3% vs. 37.9%) and treatment (24.9% vs. 29.0%) increased in middle- and low-income countries. There was a slight decrease in control (8.4% vs. 7.7%) [3]. Implementation of treatment and ensuring good pressure control constitute highly important elements in the prevention of cardiovascular and kidney diseases. Current epidemiological data in Poland, including differences between voivodeships, are important for setting regional targets for cardiovascular disease prevention and improving public health.

The aim of this study was to assess the prevalence of hypertension, its awareness, treatment, and control in individual voivodeships. The second aim was to assess the change in these parameters in the samples from individual voivodeships between 2.01.2003–31.12.2005 (WOBASZ study) and 2.01.2013–31.12.2014 in adult Poles aged 20–74 years, who were examined in the WOBASZ II study.

There are no further, newer WOBASZ surveys. The WOBASZ II study remains the most recent and largest one. Plans are in place to conduct the WOBASZ III study in 2025–2026.

Many publications are based on the results of the WOBASZ II study. However, data concerning the prevalence of hypertension, its awareness, treatment, and control in individual voivodeships has never been analyzed.

## Patients and methods

### Study design and enrollment of patients

The study methodology (study protocol, training materials, questionnaires and instructions for researchers) was based on the World Health Organization – MONItoring trends and determinants in CArdiovascular disease (WHO MONICA) project [4], 1st edition of the Multicentre National Population Health Survey (**W**ieloośrodkowe **O**gólnopolskie **Ba**danie **S**tanu **Z**drowia Ludności WOBASZ study) [5], and the guidelines of the European Health Examination Survey [6]. The study covered 16 voivodeships. Sampling had three stages, stratified by voivodeships, type of communes and sex. First, a list of communes was developed based on the Central Statistical Office’s classification of national territorial units: 1,847 small communes (population <8,000), 558 medium communes (8,000–40,000), and 102 large communes (>40,000). From each voivodeship, two small, two medium, and two large communes were selected, mirroring those included in the WOBASZ study (2003–2005). However, the individual samples drawn were independent.

A sample of 15,200 individuals aged 19 years and older (within each selected commune, 70 men and 70 women) was randomly selected from the Polish population using the individual identification number. This selection of the study sample allowed us to obtain cross-sectional and representative epidemiological data on the Polish population.

Finally, the WOBASZ study included 14755 individuals (7783 women and 6972 men), while the WOBASZ II study included 6163 patients (3406 women and 2757 men); the participation rates were 76.9% and 45.5%, respectively [7]. The WOBASZ and WOBASZ II studies were approved by the Bioethics Committee of the Institute of Cardiology in Warsaw.

Each participant of the studies signed a written informed consent to participate.

Blood pressure (BP) measurements were performed according to the Polish Society of Hypertension (Polskie Towarzystwo Nadciśnienia Tętniczego – PTNT) 2015 [8] guidelines, which comply with the recommendations of the 2013 European Society of Cardiology/European Society of Hypertension Guidelines for the management of arterial hypertension (ESC/ESH 2013) [9]. Automatic devices OMRON M-51 (OMRON Ltd, Tokyo, Japan) were used in the WOBASZ study, and AND UA-631(AND Company Ltd, Tokyo, Japan) in the WOBASZ II study. All blood pressure measurements were performed during a single visit, on the right arm, after the patient had been sitting and resting for 5 minutes, and three readings were taken at 2-minute intervals.

The analysis included mean values of the second and third measurements. In those cases where 3 measurements were not taken, one measurement or the mean of two measurements was accepted.

The following definitions were assumed (according to the World Hypertension League Expert Committee [10]):

- Prevalence of hypertension is defined as the proportion of the population with systolic blood pressure (SBP) ≥140 mm Hg or diastolic blood pressure (DBP) ≥90 mm Hg or who report currently (regularly for the last 2 weeks) taking medication for high BP, relative to whole population.
- Prevalence of awareness of hypertension is defined as the proportion of patients with hypertension who either report having been diagnosed with hypertension by a health professional or who report taking medication for high BP to hypertensive patients; (positive responses to the questions: “Have you ever been told by a doctor that you had hypertension, also called high BP?” and “Have you ever taken medicine prescribed for BP pressure?”)
- Prevalence of treatment of hypertension is defined as the proportion of patients with hypertension who report taking medication for high BP to hypertensive patients; (affirmative response to the questions: “Have you taken these medicines regularly during the last 2 weeks?”).
- Prevalence of controlled hypertension is defined as the proportion of patients with hypertension who both: report taking medication for high BP and have SBP < 140 mm Hg and DBP < 90 mm Hg to hypertensive patients

### Statistical data analysis

BP distribution was checked with the Shapiro-Wilk test (normal distribution) and was described using arithmetic means and standard deviations. Age-standardized prevalence rates with the weights for the Polish population were calculated for 31 December 2013 [11]. To compare the mean values between studies, the t-Student test was applied. The standardized values were compared using 95% confidence intervals (95%CI). To demonstrate differences in the frequency of hypertension prevalence, awareness, treatment and control in individual voivodeships, the rate ratio (RR) with 95%CI was calculated. In the voivodeship where the above index was lowest, the RR value was 1.00. In order to compare data from the WOBASZ II study with data from the first edition of the WOBASZ study, which included adults aged 20–74, we used restricted data from the WOBASZ II study for people aged 20–74. The RR with 95%CI was calculated by dividing the standardized frequency of the above listed parameters in order to evaluate the changes in the prevalence of hypertension, awareness, treatment, and control of hypertension between WOBASZ and WOBASZ II studies. RR value below 1.0 indicated a decreased prevalence, and above 1.0 an increased prevalence. The relationship between socioeconomic factors and control of the hypertension was assessed using the Spearman rank-order correlation. Statistical hypotheses were verified at a significance level of *P < 0.05*. Calculations were performed with the statistical package STATISTICA PL (data analysis software system) StatSoft, Inc. (2016) version 12.5.

## Results

In all voivodeships, the mean values for systolic blood pressure were significantly lower in women than in men (Table 1).

**Table 1:** Average age (SD) and mean values of systolic and diastolic pressure (SD) in voivodeships in the WOBASZ II study (population 19-99 years).

	Men				Women				P^a^	P^b^
	N	Age. years	SBP. mmHg	DBP. mmHg	N	Age. yeras	SBP. mmHg	DBP. mmHg		
Dolnośląskie	139	47.4 (14.8)	133.7 (16.3)	81.4 (9.5)	177	47.4 (14.4)	125.5 (17.3)	79.8 (9.6)	<0.001	0.14
Kujawsko-Pomorskie	214	46.1 (14.1)	132.9 (17)	81.5 (10)	237	47.1 (14.2)	124.2 (17.9)	78.2 (10)	<0.001	<0.001
Lubelskie	138	47.2 (14.1)	134.1 (19.7)	80.7 (10)	163	48.1 (15.2)	129 (23.2)	79.2 (9.9)	0.04	0.19
Lubuskie	124	48.8 (14.5)	137.9 (19.3)	83.5 (10.3)	134	47 (13.3)	126.8 (19.4)	80.2 (10.8)	<0.001	0.01
Łódzkie	114	45.1 (14)	132.5 (17.6)	81.9 (11.9)	145	48.3 (14.7)	126.4 (17.7)	79.5 (10.4)	0.01	0.08
Małopolskie	147	46.7 (14.4)	134.3 (16.9)	82.7 (10.6)	206	46.7 (14.8)	127.1 (19.1)	79.8 (10.5)	<0.001	0.01
Mazowieckie	174	46.6 (14.7)	131.8 (16)	82.3 (10)	195	48.1 (13.4)	126.3 (18.1)	80 (10.8)	<0.01	0.0351
Opolskie	151	46.4 (15)	135.8 (19.4)	82.1 (10.9)	207	47.4 (14.3)	128.1 (18.6)	80.5 (11.2)	<0.001	0.18
Podkarpackie	118	47.4 (14.4)	135.4 (17.5)	83.5 (11)	143	48.3 (13.7)	128.3 (18.2)	79.7 (10.3)	<0.01	<0.01
Podlaskie	202	46.9 (14.3)	131.9 (16.6)	81.7 (10.5)	252	47.1 (14.3)	123.6 (18.6)	78.3 (10.2)	<0.001	<0.001
Pomorskie	169	44.3 (14.6)	133.7 (18.2)	81.2 (10)	198	45.4 (14.3)	126.7 (19.7)	80 (10.5)	<0.001	0.27
Śląskie	159	45.6 (15.4)	132.5 (20)	80.2 (11.1)	213	47 (13.7)	126 (19.9)	79 (11.3)	<0.01	0.31
Świętokrzyskie	154	49.1 (14)	137.9 (17.8)	83.1 (10.7)	167	49 (14.2)	132.3 (19.1)	83.1 (11)	<0.01	1
Warmińsko-Mazurskie	221	47.3 (14.8)	132.5 (16.8)	81.4 (10.8)	246	47.5 (14.8)	123.9 (18.1)	77.4 (11.2)	<0.001	<0.001
Wielkopolskie	180	47.9 (15.1)	134.4 (18.6)	81.6 (12)	229	48.3 (14.2)	123.9 (19.6)	77.4 (11.6)	<0.001	<0.001
Zachodniopomorskie	171	45.7 (14)	134.5 (17.5)	81.9 (10.8)	199	47.1 (14.3)	125.2 (17.5)	78.4 (9.6)	<0.001	<0.001

^a^- SBP men vs women; ^b^- DBP men vs women

Abbreviations: SBP- systolic blood pressure; DBP – diastolic blood pressure; SD – standard deviation

In the WOBASZ II study population aged 20−74 years, we also found considerable regional differences in the prevalence of hypertension, awareness, treatment and control (Tables 2–6). In men from Lubuskie Voivodeship, hypertension occurred more frequently by 58% than in Lubelskie Voivodeship [60.12% (95%CI:60.05–60.2) vs. 38.01% (95%CI:37.98–38.05)]. In women, hypertension occurred more frequently by 70% in Świętokrzyskie Voivodeship than in Podlaskie Voivodeship [46.17% (95%CI:46.12–46.22) and vs. 29.45% (95%CI:29.42–29.47)]. Awareness of hypertension was higher by 54% among men in Lubelskie Voivodeship than in Podkarpackie Voivodeship [73.08% (95%CI:73.03–73.14) vs. 47.41% (95%CI:47.37–47.46)]; and among women it was twice as common in Mazowieckie Voivodeship than in Pomorskie Voivodeship [84.05% (95%CI:84.02–84.09) vs. 41.06% (95%CI:41.06–41.06)]. Men from Pomorskie Voivodeship were more than twice as likely to be treated as men from Podkarpackie Voivodeship [42% (95%CI:41.96–42.04) vs. 20.55% (95%CI:20.55–20.55)]. Similarly, women from Lubelskie Voivodeship were treated over two-fold more frequently than women from Pomorskie Voivodeship [55.29% (95%CI:55.24–55.34) vs. 25.56% (95%CI:25.56–25.56)]. In men from Pomorskie Voivodeship, BP control was almost six times better than in Podkarpackie Voivodeship [26.64%; (26.62–26.67) vs. 5.11%; (95%CI:5.11–5.11)]. BP normalization was achieved more than four times more frequently in women from Lubelskie Voivodeship than in Pomorskie Voivodeship [37.63%; (95%CI:37.59–37.67) vs. 9.25%; (95%CI:9.25–9.25)].

**Table 2:** Age-standardized prevalence of hypertension in a subsample at age restricted to 20-74 years by voivodeship in WOBASZ Studies.

Voivodeship	WOBASZ % (95%CI)	WOBASZ II % (95%CI)	*P*
Dolnośląskie			
Men	47.52 (47.48-47.56)	49.87 (49.83-49.9)	0.63
Women	39.86 (39.83-39.9)	39.25 (39.22-39.28)	0.89
Kujawsko-pomorskie			
Men	42.97 (42.93-43.02)	49.33 (49.28-49.37)	0.12
Women	38.57 (38.54-38.6)	36.43 (36.4-36.47)	0.58
Lubelskie			
Men	32.63 (32.6-32.67)	38.01 (37.98-38.05)	0.23
Women	35.98 (35.95-36.02)	42.09 (42.06-42.13)	0.16
Lubuskie			
Men	47.37 (47.31-47.43)	60.12 (60.05-60.2)	0.01
Women	42.01 (41.96-42.07)	45.74 (45.68-45.79)	0.45
Łódzkie			
Men	36.56 (36.53-36.6)	46.39 (46.35-46.43)	0.05
Women	32.66 (32.63-32.69)	44.05 (44.03-44.07)	0.01
Małopolskie			
Men	48.21 (48.17-48.25)	43.22 (43.18-43.25)	0.3
Women	41.66 (41.63-41.69)	36.29 (36.26-36.32)	0.19
Mazowieckie			
Men	54.47 (54.44-54.5)	57.22 (57.18-57.25)	0.53
Women	45.16 (45.13-45.18)	40.94 (40.92-40.97)	0.31
Opolskie			
Men	52.34 (52.27-52.41)	53.03 (52.97-53.1)	0.89
Women	41.02 (40.97-41.07)	45.44 (45.38-45.5)	0.29
Podkarpackie			
Men	46.16 (46.11-46.2)	51.1 (51.06-51.15)	0.34
Women	36.49 (36.45-36.52)	37.68 (37.64-37.71)	0.8
Podlaskie			
Men	43.42 (43.36-43.48)	41.11 (41.05-41.17)	0.59
Women	37.44 (37.39-37.48)	29.45 (29.42-29.47)	0.03
Pomorskie			
Men	44.12 (44.08-44.17)	47.45 (47.4-47.49)	0.46
Women	40.89 (40.85-40.93)	37.69 (37.66-37.72)	0.44
Śląskie			
Men	48.87 (48.84-48.9)	46.51 (46.48-46.53)	0.61
Women	43.74 (43.71-43.77)	41.92 (41.89-41.95)	0.66
Świętokrzyskie			
Men	39.74 (39.69-39.79)	51.78 (51.72-51.84)	0.01
Women	34.69 (34.66-34.73)	46.17 (46.12-46.22)	0.09
Warmińsko-mazurskie			
Men	45.3 (45.24-45.35)	46.34 (46.29-46.4)	0.8
Women	43.48 (43.43-43.53)	36.04 (36.01-36.07)	0.05
Wielkopolskie			
Men	51.56 (51.53-51.6)	50.05 (50.02-50.09)	0.73
Women	39.91 (39.88-39.94)	34.79 (34.77-34.82)	0.18
Zachodniopomorskie			
Men	42.59 (42.55-42.64)	50.71 (50.66-50.76)	0.07
Women	41.78 (41.73-41.82)	42.12 (42.08-42.16)	0.93

**Table 3:** Age-standardized prevalence of awareness of hypertension in a subsample at age restricted to 20-74 years by voivodeship in WOBASZ Studies.

Voivodeship	WOBASZ % (95%CI)	WOBASZ II % (95%CI)	*P*
Dolnośląskie			
Men	57.99 (57.95-58.04)	64.78 (64.74-64.83)	0.31
Women	82.57 (82.52-82.62)	72.14 (72.1-72.19)	0.06
Kujawsko-pomorskie			
Men	52.51 (52.47-52.56)	54.54 (54.49-54.59)	0.73
Women	69.44 (69.39-69.5)	57.33 (57.3-57.37)	0.05
Lubelskie			
Men	80.06 (80.01-80.12)	73.08 (73.03-73.14)	0.25
Women	91.39 (91.33-91.45)	82.08 (82.03-82.13)	0.03
Lubuskie			
Men	61.3 (61.23-61.37)	68.73 (68.66-68.8)	0.24
Women	70.27 (70.19-70.35)	62.89 (62.82-62.96)	0.28
Łódzkie			
Men	73.27 (73.22-73.32)	49.72 (49.68-49.76)	<0.001
Women	81.6 (81.54-81.65)	72.36 (72.32-72.41)	0.11
Małopolskie			
Men	56.51 (56.47-56.55)	59.98 (59.94-60.02)	0.61
Women	88.43 (88.38-88.47)	69.68 (69.63-69.72)	<0.001
Mazowieckie			
Men	54.87 (54.84-54.9)	63.03 (63-63.06)	0.15
Women	76.59 (76.55-76.62)	84.05 (84.02-84.09)	0.14
Opolskie			
Men	63.6 (63.53-63.68)	57.56 (57.48-57.63)	0.34
Women	68.85 (68.78-68.93)	64.07 (63.99-64.14)	0.42
Podkarpackie			
Men	57.83 (57.78-57.88)	47.41 (47.37-47.46)	0.14
Women	67.67 (67.61-67.72)	73.66 (73.61-73.72)	0.38
Podlaskie			
Men	63.13 (63.06-63.2)	68.22 (68.15-68.3)	0.40
Women	75.11 (75.03-75.18)	52.32 (52.32-52.32)	<0.001
Pomorskie			
Men	60.05 (60-60.09)	58.65 (58.6-58.69)	0.83
Women	78.41 (78.35-78.46)	41.06 (41.06-41.06)	<0.001
Śląskie			
Men	70.43 (70.39-70.47)	52.29 (52.27-52.32)	0.01
Women	68.99 (68.96-69.03)	58.1 (58.07-58.14)	0.08
Świętokrzyskie			
Men	60.4 (60.33-60.46)	51.47 (51.41-51.53)	0.16
Women	65.7 (65.64-65.77)	76.96 (76.88-77.03)	0.07
Warmińsko-Mazurskie			
Men	63.47 (63.41-63.53)	66.11 (66.05-66.18)	0.64
Women	79.26 (79.19-79.33)	69.92 (69.85-69.99)	0.07
Wielkopolskie			
Men	50.76 (50.73-50.8)	59.52 (59.48-59.56)	0.14
Women	64.67 (64.62-64.71)	73.22 (73.17-73.26)	0.14
Zachodniopomorskie			
Men	71.74 (71.68-71.8)	59.26 (59.2-59.32)	0.04
Women	77.31 (77.25-77.37)	75.45 (75.39-75.51)	0.73

**Table 4:** Age-standardized prevalence of treatment of hypertension in a subsample at age restricted to 20-74 years by voivodeship in WOBASZ Studies.

Voivodeship	WOBASZ % (95%CI)	WOBASZ II % (95%CI)	*P*
Dolnośląskie			
Men	19.11 (19.11-19.11)	33.94 (33.94-33.94)	0.01
Women	34.91 (34.91-34.91)	46.69 (46.66-46.73)	0.08
Kujawsko-Pomorskie			
Men	24.57 (24.54-24.6)	32.95 (32.92-32.99)	0.11
Women	28.72 (28.72-28.72)	33.35 (33.34-33.35)	0.43
Lubelskie			
Men	26.16 (26.16-26.16)	37.7 (37.66-37.74)	0.09
Women	35.32 (35.32-35.32)	55.29 (55.24-55.34)	0.01
Lubuskie			
Men	21.48 (21.44-21.52)	28.32 (28.32-28.32)	0.21
Women	26.95 (26.91-26.99)	47.07 (47.07-47.07)	0.01
Łódzkie			
Men	27.12 (27.1-27.14)	33.24 (33.24-33.24)	0.38
Women	37.63 (37.63-37.64)	31.58 (31.58-31.58)	0.37
Małopolskie			
Men	17.94 (17.93-17.96)	31.59 (31.57-31.62)	0.02
Women	35.43 (35.4-35.46)	29.7 (29.7-29.7)	0.36
Mazowieckie			
Men	23.07 (23.06-23.09)	30.43 (30.41-30.45)	0.14
Women	31.61 (31.61-31.62)	35.25 (35.25-35.25)	0.53
Opolskie			
Men	25.07 (25.04-25.1)	32.71 (32.67-32.76)	0.19
Women	24.67 (24.67-24.67)	44.43 (44.38-44.49)	<0.001
Podkarpackie			
Men	19.61 (19.61-19.61)	20.55 (20.55-20.55)	0.87
Women	29.25 (29.25-29.25)	32.4 (32.4-32.4)	0.64
Podlaskie			
Men	26.16 (26.13-26.19)	32.84 (32.8-32.88)	0.25
Women	32.67 (32.67-32.67)	31.47 (31.47-31.48)	0.85
Pomorskie			
Men	21.88 (21.85-21.91)	42 (41.96-42.04)	<0.001
Women	28.9 (28.9-28.9)	25.56 (25.56-25.56)	0.58
Śląskie			
Men	24.09 (24.07-24.11)	28.8 (28.8-28.8)	0.42
Women	26.53 (26.51-26.55)	34.09 (34.09-34.09)	0.20
Świętokrzyskie			
Men	26.16 (26.12-26.19)	29.57 (29.52-29.61)	0.56
Women	26.95 (26.95-26.95)	34.99 (34.99-34.99)	0.19
Warmińsko-Mazurskie			
Men	16.77 (16.74-16.8)	40.34 (40.29-40.39)	<0.001
Women	33.21 (33.18-33.24)	42.01 (42.01-42.01)	0.13
Wielkopolskie			
Men	22.28 (22.26-22.3)	35.37 (35.34-35.4)	0.01
Women	24.16 (24.14-24.17)	32.79 (32.79-32.79)	0.12
Zachodniopomorskie			
Men	30.06 (30.02-30.1)	32.9 (32.86-32.94)	0.63
Women	36.52 (36.49-36.56)	48.19 (48.14-48.24)	0.07

**Table 5:** Age-standardized prevalence of control hypertension in a subsample at age restricted to 20-74 years by voivodeship in WOBASZ Studies.

Voivodeship	WOBASZ % (95%CI)	WOBASZ II % (95%CI)	*P*
Dolnośląskie			
Men	3.56 (3.56-3.56)	12.07 (12.07-12.07)	0.01
Women	6.99 (6.98-6.99)	29.71 (29.68-29.74)	<0.001
Kujawsko-Pomorskie			
Men	5.53 (5.53-5.53)	18.61 (18.58-18.64)	<0.001
Women	6.25 (6.25-6.25)	18.38 (18.38-18.38)	0.01
Lubelskie			
Men	6.57 (6.57-6.57)	17.94 (17.94-17.94)	0.01
Women	15.54 (15.54-15.54)	37.63 (37.59-37.67)	<0.001
Lubuskie			
Men	4.51 (4.51-4.51)	13.45 (13.45-13.45)	0.01
Women	5.63 (5.63-5.63)	27.45 (27.45-27.45)	<0.001
Łódzkie			
Men	4.27 (4.27-4.27)	16.2 (16.2-16.2)	0.01
Women	8.23 (8.23-8.23)	18.77 (18.77-18.77)	0.02
Małopolskie			
Men	9.69 (9.67-9.7)	16.92 (16.9-16.94)	0.1
Women	17.04 (17.02-17.06)	9.57 (9.56-9.57)	0.11
Mazowieckie			
Men	7.23 (7.22-7.24)	17.45 (17.43-17.46)	0.01
Women	14.87 (14.87-14.87)	15.33 (15.33-15.33)	0.92
Opolskie			
Men	5.74 (5.74-5.75)	12.23 (12.23-12.23)	0.07
Women	5.25 (5.25-5.26)	15.74 (15.74-15.74)	0.01
Podkarpackie			
Men	7.11 (7.1-7.11)	5.11 (5.11-5.11)	0.57
Women	6.53 (6.53-6.53)	16.04 (16.04-16.04)	0.02
Podlaskie			
Men	7.3 (7.3-7.3)	14.3 (14.3-14.3)	0.06
Women	11.03 (11.03-11.03)	18.74 (18.74-18.74)	0.09
Pomorskie			
Men	7.57 (7.57-7.57)	26.64 (26.62-26.67)	<0.001
Women	6.18 (6.18-6.18)	9.25 (9.25-9.25)	0.37
Śląskie			
Men	8.75 (8.73-8.76)	14.57 (14.57-14.57)	0.15
Women	9.55 (9.54-9.56)	15.06 (15.06-15.06)	0.18
Świętokrzyskie			
Men	8.46 (8.46-8.46)	13.11 (13.08-13.14)	0.23
Women	8.16 (8.16-8.16)	18.98 (18.97-18.98)	0.01
Warmińsko-Mazurskie			
Men	5.71 (5.71-5.71)	18.02 (18.02-18.02)	<0.001
Women	9.3 (9.3-9.3)	26.08 (26.08-26.08)	<0.001
Wielkopolskie			
Men	3.54 (3.54-3.54)	13.28 (13.28-13.29)	<0.001
Women	6.13 (6.13-6.13)	20.59 (20.59-20.59)	<0.001
Zachodniopomorskie			
Men	7.9 (7.9-7.91)	21.7 (21.67-21.73)	<0.001
Women	10.4 (10.4-10.4)	36.48 (36.43-36.52)	<0.001

**Table 6:** Age-adjusted relative risks of hypertension prevalence, awareness, treatment, and control by voivodeship in the WOBASZ II population aged 20–74 years (voivodeship with the lowest value as a reference).

	Prevalance		Awareness		Treatment		Control	
	Men	Women	Men	Women	Men	Women	Men	Women
Dolnośląskie	1.32 (0.86-2.01)	1.44 (0.96-2.17)	1.37 (0.69-2.71)	1.78 (0.89-3.54)	1.67 (0.77-3.62)	1.86 (0.93-3.73)	2.6 (0.67-10.05)	**3.27 (1.3-8.25)**
Kujawsko-Pomorskie	1.3 (0.86-1.98)	1.33 (0.91-1.95)	1.14 (0.62-2.11)	1.41 (0.76-2.62)	1.64 (0.79-3.38)	1.35 (0.69-2.65)	**3.94 (1.12-13.83)**	2.01 (0.78-5.13)
Lubelskie	1.00	**1.55 (1.02-2.35)**	1.54 (0.73-3.26)	2.02 (0.95-4.29)	1.92 (0.86-4.27)	**2.22 (1.11-4.43)**	**3.97 (1.05-15.03)**	**4.11 (1.66-10.19)**
Lubuskie	**1.58 (1.03-2.42)**	**1.67 (1.08-2.59)**	1.44 (0.74-2.82)	1.53 (0.77-3.03)	1.45 (0.67-3.11)	1.88 (0.92-3.83)	2.87 (0.77-10.76)	**3.05 (1.18-7.89)**
Łódzkie	1.23 (0.81-1.86)	**1.62 (1.05-2.49)**	1.03 (0.5-2.13)	1.77 (0.88-3.56)	1.7 (0.74-3.9)	1.24 (0.59-2.57)	3.68 (0.94-14.37)	2.08 (0.78-5.56)
Małopolskie	1.14 (0.75-1.74)	1.33 (0.9-1.98)	1.26 (0.64-2.49)	1.69 (0.88-3.27)	1.57 (0.71-3.46)	1.19 (0.59-2.4)	**3.71 (1-13.83)**	1.07 (0.37-3.12)
Mazowieckie	**1.51 (1.01-2.26)**	**1.5 (1.01-2.23)**	1.33 (0.71-2.5)	**2.07 (1-4.3)**	1.54 (0.74-3.21)	1.42 (0.72-2.79)	**3.75 (1.06-13.29)**	1.69 (0.64-4.43)
Opolskie	1.4 (0.91-2.14)	**1.67 (1.13-2.46)**	1.2 (0.62-2.3)	1.56 (0.84-2.88)	1.61 (0.75-3.44)	1.78 (0.93-3.42)	2.58 (0.68-9.79)	1.75 (0.68-4.51)
Podkarpackie	1.35 (0.89-2.07)	1.38 (0.89-2.14)	1 (0.5-1.99)	1.82 (0.88-3.77)	1.00	1.29 (0.61-2.71)	1.00	1.75 (0.62-4.91)
Podlaskie	1.09 (0.71-1.67)	1.00	1.44 (0.74-2.78)	1.29 (0.68-2.41)	1.63 (0.77-3.46)	1.27 (0.63-2.55)	3.16 (0.86-11.61)	1.99 (0.76-5.18)
Pomorskie	1.24 (0.82-1.89)	1.39 (0.93-2.07)	1.24 (0.64-2.38)	1 (0.52-1.91)	**2.11 (1-4.45)**	1.00	**5.74 (1.63-20.19)**	1.00
Śląskie	1.22 (0.8-1.86)	**1.53 (1.04-2.26)**	1.1 (0.57-2.13)	1.42 (0.76-2.65)	1.41 (0.64-3.08)	1.36 (0.69-2.69)	3.06 (0.81-11.47)	1.6 (0.6-4.25)
Świętokrzyskie	1.36 (0.88-2.11)	**1.7 (1.13-2.57)**	1.07 (0.56-2.04)	1.89 (0.96-3.72)	1.48 (0.69-3.16)	1.38 (0.7-2.73)	2.95 (0.8-10.94)	2.12 (0.83-5.44)
Warmińsko-Mazurskie	1.22 (0.79-1.87)	1.32 (0.9-1.93)	1.39 (0.75-2.6)	1.72 (0.92-3.21)	2.04 (1-4.16)	1.68 (0.87-3.23)	**3.83 (1.09-13.46)**	**2.82 (1.15-6.92)**
Wielkopolskie	1.32 (0.88-1.98)	1.28 (0.87-1.89)	1.25 (0.66-2.33)	1.81 (0.94-3.47)	1.78 (0.86-3.7)	1.32 (0.67-2.6)	2.79 (0.76-10.2)	2.27 (0.9-5.73)
Zachodnio-Pomorskie	1.33 (0.87-2.05)	**1.54 (1.04-2.28)**	1.24 (0.65-2.36)	1.84 (0.95-3.58)	1.65 (0.78-3.48)	1.91 (0.98-3.72)	**4.76 (1.35-16.79)**	**3.95 (1.62-9.62)**

We conducted an additional analysis to look for differences. Among other things, we took into account the statistical Local Human Development Index (LHDI) published by the Central Statistical Office, along with its individual components: HI (health index), EI (education index), and WI (wealth index). After analyzing the LHDI and its components (HI, EI, and WI), the number of outpatient healthcare consultations per capita, the number of unemployed individuals, the number of doctors and nurses per 10,000 residents, and GDP per capita, we were unable to find an explanation for the prevalence of hypertension, its awareness, treatment, and control in each province. However, we found a significant inverse relationship between the health index (HI) and the prevalence of hypertension (R Pearson = −0.665603; p < 0.01). Additionally, we found no correlation between parameters describing the epidemiology of hypertension and place of residence (size of communes). Comprehensive data are not shown in this publication.

### Change within a decade – comparison of the WOBASZ and WOBASZ II study results

**Fig 1:**
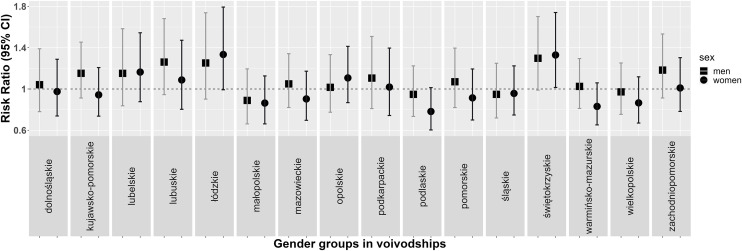
Rate ratios (95% confidence intervals [CIs]) of the prevalence of hypertension in a subsample at age restricted to 20-74 years in men and women by voivodeship in WOBASZ studies.

A comparison of the WOBASZ I and WOBASZ II studies led to the conclusion that the greatest increase in the prevalence of hypertension in men occured in Świętokrzyskie Voivodeship (39.7% vs. 51.8%) and in women from Łódzkie Voivodeship (32.7% vs. 44%), while the largest decrease was observed in men from Małopolskie Voivodeship (48.2% vs. 43.2%) and in women (37.4% vs. 29.4%) from Podlaskie Voivodeship (Fig 1).

**Fig 2:**
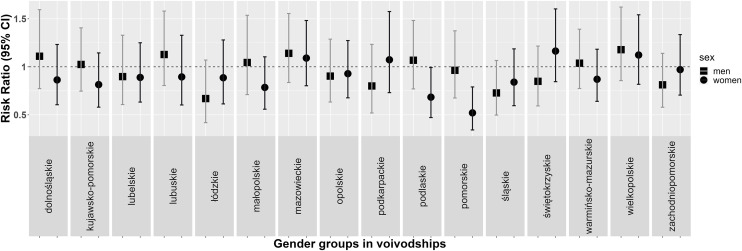
Rate ratios (95% confidence intervals [CIs]) of the prevalence of awareness in a subsample at age restricted to 20-74 years in men and women by voivodeship in WOBASZ studies.

The greatest increase in awareness of hypertension was observed in men from Wielkopolskie Voivodeship (50.8% vs. 59.5%) and in women from Świętokrzyskie Voivodeship (65.7% vs. 77%) and the greatest decrease in awareness was observed in men from Łódzkie Voivodeship (73.3% vs. 49.7%) and in women from Pomorskie Voivodeship (78.4% vs. 41.1%) (Fig 2).

**Fig 3:**
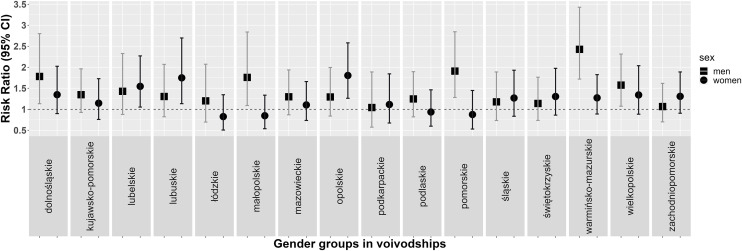
Rate ratios (95% confidence intervals [CIs]) of the prevalence of treatment in a subsample at age restricted to 20-74 years in men and women by voivodeship in WOBASZ studies.

**Fig 4:**
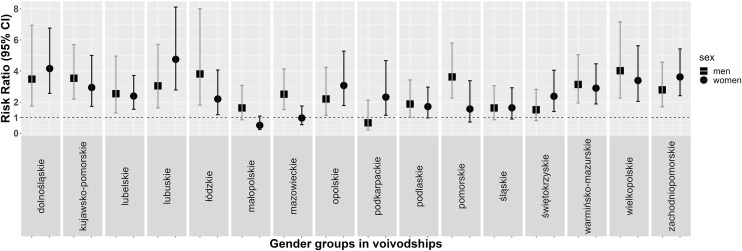
Rate ratios (95% confidence intervals [CIs]) of the prevalence of control of hypertension in a subsample at age restricted to 20-74 years in men and women by voivodeship in WOBASZ studies.

The largest increase in the percentage of people treated for hypertension was observed in men from Warmińsko-Mazurskie Voivodeship (16.8% vs. 40.3%) and in women from Opolskie Voivodeship (24.7% vs. 44.4%). The largest decrease in the number of treated patients was observed in women from Łódzkie Voivodeship (37.6% vs. 31.6%) (Fig 3). The greatest increase in the BP control was observed in men from Wielkopolskie Voivodeship (3.5% vs. 13.3%) and in women from Lubuskie Voivodeship (5.6% vs. 27.4%). The greatest decrease in the BP control was observed in men from Podkarpackie Voivodeship (7.1% vs. 5.1%) and in women from Małopolskie Voivodeship (17% vs. 9.6%) (Fig 4).

## Discussion

This study is the first to compare the results of two Polish cross-sectional epidemiological studies that assessed changes in the prevalence, awareness, treatment and control of hypertension in all 16 voivodeships over a 10-year period.

The current nationwide epidemiological information on arterial hypertension in the age group of 20–74 years and in the elderly (i.e., over 74 years) has been published previously [12,13] in a similar style as for other risk factors for cardiovascular disease, i.e., obesity, hyperlipidemia, metabolic syndrome, low physical activity, and tobacco smoking [14–21], and has been a subject of a comprehensive review [22].

In all voivodeships, lower systolic and diastolic BP was found in women than in men. This remains in line with the worldwide findings. In the WOBASZ II sample, both the systolic and diastolic blood pressure were higher than the world average, which in 2015 was 127.0 (95%CI:125.7–128.3) mmHg for SBP and 78.7 (95%CI:77.9–79.5) mmHg for DBP in men and 122.3 (95%CI:121.0–123.6) mmHg for SBP and 76.7 (75.9–77.6) mmHg for DBP in women [23].

The differences in the frequency of BP control between voivodeships were also demonstrated in the large-scale Polish cohort study on hypertension control and resistant hypertension – Pol-Fokus study, with the worst BP control in Warmińsko-Mazurskie Voivodeship and the best in Mazowieckie Voivodeship. Due to the different method of patient selection in the Pol-Fokus study, the results for BP control in individual voivodeships were significantly different from those in the WOBASZ II study[24].

We have a small amount of data describing the prevalence of hypertension in relation to the region of residence of individual countries. The German population is undoubtedly very similar to the Polish population in terms of geography and culture. This population also shows considerable regional differences in the distribution of social and economic factors influencing the epidemiology of arterial hypertension [25]. In the population of the DEGS1 study, similar to the WOBASZ II study, significant differences between regions were found for both men and women. The maximum difference for hypertension prevalence [OR (odds ratio) men/women DEGS1 vs. RR men/women WOBASZ II] was 1.42/2.07 vs. 1.61/1.53, for awareness 4.35/2.37 vs. 1.49/2.21, for treatment 2.42/1.32 vs. 2.34/2.59 and for control 1.69/1.35 vs. 5.9/5.21 [25]. A slightly lower regional variation of the prevalence of hypertension and its treatment was found in Switzerland. The lowest frequency of hypertension diagnosis was found in the central region – 24.3%, while the highest frequency was found in the north-western region – 30%. On the other hand, the highest percentage of treated persons was found in the southern region – 66.3% and the lowest in the eastern region – 57.3% [26]. The citizens of Luxembourg were also found to exhibit geographic differences, and the highest OR for hypertension prevalence was determined in the most industrial south-west region [OR:1.2; (95%CI: 0.9–1.6)] [27]. In the Croatian Adult Health Survey of 2003, hypertension was found in 40.5% of men and in 34.9% of women, and no significant differences were observed according to the region of residence, except the prevalence in men in the Northern and Eastern parts of Croatia [28]. In China, a country with a high variability of socioeconomic factors influencing the risk of cardiovascular disease, large differences related to the place of residence of the examined persons have also been demonstrated. Thus, in the Chinese part of the Prospective Urban Rural Epidemiology (PURE) Study [29], the highest prevalence of hypertension was observed in the eastern regions – 44.3%, medium in central China – 39.3%, and the lowest in the western regions – 37.0%. The highest awareness was observed in the central provinces – 44.3%, and the lowest in the west – 37.0%. Similarly, the highest frequency of implemented antihypertensive treatment was found in the central regions – 37.7%, and the lowest in the west – 26.7%. On the other hand, the highest percentage of controlled hypertension was found in the east, and the lowest in the western regions – 7.1% [30]. In a meta-analysis of 47 studies conducted in China in the period between 2002 and 2012 and involving 153481 residents, regional differences in the prevalence of hypertension were confirmed ranging from 28.3% to 30.4% in the northern provinces to 16.2% in the mid-eastern provinces [31].

The international literature provides very limited information on the causes for regional variations within one country. Taking Austria and Great Britain as an instance, variances in hypertension prevalence persist, notwithstanding adjustments for numerous risk determinants like smoking, dietary habits, physical activity, educational background, social support structures, and ethnic origin. The researchers postulated that an in-depth exploration of additional influences, encompassing lifestyle choices, personal attitudes, value systems, and cultural dynamics, is imperative for elucidating these persisting discrepancies. Complementary investigations also propose the necessity of probing into biological, environmental, and potential genetic underpinnings [32,33].

Previous studies describing the impact of socioeconomic factors on cholesterol control, obesity prevalence, and physical activity levels in the entire population in the WOBASZ II study showed a significant correlation with level of education, smoking, coexisting diabetes and other CVDs, and frequent doctor visits (>=4). As in the general population, the observed differences between provinces may have been influenced by access to medical services, particularly primary care, and level of education [12,15,19,20]. In this study, we did not perform such analyses due to the small size of the subgroups. In another of our publications, we evaluated the effect of age, gender, BMI, diabetes, hyperlipidemia, physical activity, education, and comorbidities on blood pressure control in the general population [21].

Considerable regional differences were also demonstrated for the prevalence of obesity in the Polish population included in the WOBASZ and WOBASZ II studies [14].

The results of the WOBASZ II study could serve as a valuable guide for primary care. They could indicate which provinces should increase the number of hypertension screening tests and which should intensify treatment.

The Polish guidelines for diagnosing and treating hypertension refer to the results of the WOBASZ and WOBASZ II studies. They also set antihypertensive therapy targets that align with the ESC recommendations. While these guidelines provide detailed descriptions of the necessary diagnostic tests and propose straightforward treatment regimens for hypertension, they lack a public health strategy to improve hypertension control in Poland. Therefore, we propose a paradigm shift in the treatment of modifiable risk factors for cardiovascular disease, including hypertension. To improve compliance with hypertension treatment, we propose introducing a “success fee.” After achieving control of a modifiable CVD risk factor for a patient, the patient’s tax or health insurance contributions would be reduced, and the physician would receive a financial reward [35].

### Limitations

The weak point of the study is the fact that pressure measurements were performed only at one visit. This may have led to an overestimation of the prevalence of hypertension in Poland and an underestimation of its awareness, treatment frequency and control. Another weak point of the presented study is the relatively low participation rate, which was 45.5% compared to 70.5% in the previous study. In recent years, epidemiological studies conducted in Western Europe have also shown a decrease in the participation rate, for example in the EHES (European Health Examination Survey Pilot Project 2009–2012) it ranged from 31% to 74% in women and from 16% to 57% in men [34]. In the WOBASZ II study, no significant correlation was observed between the prevalence, awareness, treatment and control of hypertension and the participation rate in the individual voivodeships. The possible influence of the participation rate and the conduct of measurements during only one visit has been discussed in another paper [12].

One of the strengths of WOBASZ I and II is the large number of people examined (n = 14769 in the first study, which was completed in 2005, and n = 6170 in the second). These are the two largest Polish epidemiological studies on arterial hypertension. For comparison, the NATPOL study completed in 2011 included only 2404 Polish residents. The selection of the examined group was performed appropriately – a layered random sample was used, and an experienced and trained team participated in both editions of the WOBASZ study, which enabled representative results to be obtained from all voivodeships.

## Conclusions

Differences in prevalence, as well as awareness, treatment, and control were found in individual provinces. The prevalence of hypertension is increased in the majority of samples from Polish voivodeships.

Despite the observed improvement over the past 10 years, HT remains undiagnosed in a large number of patients, treatment is not implemented, and blood pressure control is inadequate. The data from the WOBASZ studies will allow future prevention programmes to be better tailored to the needs of different regions.

## Supporting information

S1 Data Data English PlosOne. (XLSX)
